# Healthy outcomes of patients with COVID-19 two years after the infection: a prospective cohort study

**DOI:** 10.1080/22221751.2022.2133639

**Published:** 2022-11-04

**Authors:** Dapeng Li, Xuejiao Liao, Zhi Liu, Zhenghua Ma, Jingke Dong, Guoqin Zheng, Mei Zi, Fang Wang, Qing He, Guobao Li, Zheng Zhang, Lei Liu

**Affiliations:** a Institute for Hepatology, National Clinical Research Center for Infectious Disease, Shenzhen Third People’s Hospital; The Second Affiliated Hospital, School of Medicine, Southern University of Science and Technology, Shenzhen, People’s Republic of China; b Shenzhen Research Center for Communicable Disease Diagnosis and Treatment of Chinese Academy of Medical Science, Shenzhen, People’s Republic of China; c Guangdong Key Laboratory for Anti-infection Drug Quality Evaluation, Shenzhen, People’s Republic of China

**Keywords:** COVID-19, SARS-CoV-2, long COVID, follow-up, risk factor

## Abstract

The long-term effect of coronavirus disease 2019 (COVID-19) has been rarely known. This study aimed to investigate healthy outcomes of COVID-19 survivors up to 2 years after the infection. A total of 155 COVID-19 patients, who were discharged from Shenzhen Third People's Hospital from February 2020 to April 2020, were enrolled and followed up until March 4, 2022. COVID-19 survivors received questionnaires of long COVID symptoms and psychological symptoms, pulmonary function tests, chest computed tomography (CT) scans and routine laboratory tests. Two years after infection, 36.6% of patients had at least one symptom of long COVID. Vision impairment and fatigue were the most common symptom. 35.0% of participants still had at least one psychological symptom of anxiety, depression, post-traumatic stress symptoms, and sleep difficulties. Radiographic abnormalities were presented in 50.7% of patients, with the most common features of fibrosis-like lesions and residual ground-glass opacity. Diffuse dysfunction (24.0%) was the main abnormalities of pulmonary function tests. Most laboratory parameters returned to normal range, while persistent abnormalities in kidney and liver function test were observed in a subset of participants after discharge. Two years after COVID-19 infection, persistent symptoms of long COVID and psychological symptoms, as well as abnormalities in pulmonary function tests and CT, were still common in a subset of recovering individuals. These findings were limited by the lack of a healthy control group and pre-COVID assessments, which should be confirmed by further large-scale studies.

## Introduction

As of July 29, 2022, an estimated 572 million cumulative cases and 6.4 million deaths worldwide have been attributed to COVID-19 [1]. As the world grapples with waves of infections sparked by virus variants, an emerging challenge for public health is the long-term effects of COVID-19. Symptoms can last for months or even years in a subset of COVID-19 patients, which are defined as long COVID, post-COVID syndrome, or post-acute sequelae of COVID-19 (PASC) [2–4]. Long COVID involved multiple organs, with the predominant manifestations of pulmonary, neuropsychiatric, cardiovascular, and gastrointestinal systems. Most studies have reported various health effects of COVID-19 within one year after acute infection [5–10]. A recent Wuhan study suggested that COVID-19 survivors had longitudinal improvements; however, some still had more general symptoms and psychological symptoms at two years [11]. Long COVID may contribute a huge burden on morbidity, quality of life, and healthcare costs in the coming years. More researches on long COVID were still urgently needed to better understand the long-term impact of post-COVID illness and risk factors.

The aim of this study was to investigate the health outcomes of long COVID, respiratory function, and laboratory parameters in COVID-19 survivors up to two years after the initial infection and to explore dynamic changes in those long-term health statuses.

## Methods

### Study design and participants

We conducted a single-centre prospective uncontrolled cohort study, which enrolled patients with confirmed COVID-19 admitted to Shenzhen Third People's Hospital between January 15 and April 16, 2020. All patients with COVID-19 were confirmed by the SARS-CoV-2 polymerase chain reaction (PCR) test, according to the “Clinical Guidelines for the Diagnosis and Treatment of COVID-19” (7th Edition) issued by the National Health Commission of China. Informed consent was obtained from all participants. The study protocol was approved by the Ethics Committee of Shenzhen Third People's Hospital (IRB 2020-021-02).

### Data collection and follow-up

Eligible patients were invited to participate in the study by telephone. The 2-year follow-up was performed in an outpatient setting until March 4, 2022. The acute phase was defined as the period of hospitalization following a positive test of SARS-CoV-2 infection, and the recovery phase was defined as the period after discharge. Data on patient sociodemographic and clinical characteristics were queried from the electronic medical record system. SARS-CoV-2 infected patients were classified into non-severe or severe group according to the severity of symptoms during hospitalization: non-severe groups including mild illness (patients with mild symptoms and without radiological evidence) and moderate illness (patients with fever, respiratory tract symptoms, and radiological evidence of confirmed pneumonia); severe group including severe illness (patients with respiratory distress [≥ 30 breaths/ min]; or oxygen saturation ≤ 93% at rest; or arterial partial pressure of oxygen / fraction of inspired oxygen ≤ 300mmHg) and critical illness (patients respiratory failure requiring mechanical ventilation; or shock; or other organ failure that requires intensive care unit) [12,13]. All discharged patients followed uniform discharge criteria: no fever for three consecutive days, improvement of respiratory symptoms, significant recovery of acute lung lesions, and two negative SARS-CoV-2 tests within 24 h.

### Long COVID symptoms and psychological symptoms

Long COVID, which was defined as new and persistent symptoms and more severe symptoms than COVID-19 onset, was investigated by a well-trained nurse using a structured questionnaire. The severity of dyspnea was measured using the modified Medical Research Council dyspnea scale (mMRC), with a range of 0–4. Four questionnaires were used to investigate anxiety symptoms with the Generalized Anxiety Disorder 7-item (GAD-7) scale, depression symptoms with the Patient Health Questionnaire-9 (PHQ-9), post-traumatic stress symptoms (PTSS) with the Post-traumatic Stress Disorder Checklist (PCL-5), and sleep disorders with the Pittsburgh Sleep Quality Index (PSQI).

### Pulmonary function test

The pulmonary function test was measured using a ﬂow spirometer, and diffusing capacity for carbon monoxide (DLCO) was measured using the single breath method. Pulmonary function parameters which were expressed as a percentage of the predicted value, included forced vital capacity (FVC), forced expiratory volume in one second (FEV1), FEV1/FVC, DLCO, total lung capacity (TLC), and residual volume (RV). Diffusion dysfunction was defined as the DLCO < 80% of predicted.

### Chest CT scan

Patients were scanned using a μCT 760 scanner (United Imaging, Shanghai, China), with the parameter of tube voltage 120 kV, automatic tube 40 mA, and reconstructed layer thickness 0.625 mm. The following chest CT scan features were recorded: ground-glass opacity (GGO), crazy paving, consolidation, air trapping, and fibrosis-like lesions (parenchymal band, reticulation, or traction bronchiectasis). A semiquantitative score was used to quantify the severity of lung involvement of each lobe [14,15]: 0, no involvement; 1, <5% involvement; 2, 5–25% involvement; 3, 26–49% involvement; 4, 50–75% involvement; and 5, > 75% involvement. The total CT severity score (range, 0–25) was the sum of the scores for five lobes. The CT image of these participants at admission, discharge, 3, 6, and 12 months was also collected to analyze dynamic changes.

### Laboratory parameters

Routine laboratory parameters, such as white blood cells, lymphocyte count, platelet count, haemoglobin, C-reactive protein (CRP), creatine kinase, lactate dehydrogenase (LDH), glucose, and D-dimer, and parameters of kidney and liver function, were tested. Kidney function was evaluated with a blood test (Blood urea nitrogen [BUN], estimated glomerular filtration rate [eGFR], and serum creatinine [Cr]), and urinalysis. The eGFR was calculated based on the Chronic Kidney Disease Epidemiology Collaboration (CKD-EPI) formula. Liver function tests included aspartate aminotransferase (AST), alanine aminotransferase (ALT), gamma-glutamyltransferase (GGT), and albumin. Dynamic assessments of those laboratory parameters were performed based on data of admission, discharge, 3, 6, 12, and 24 months after the infection.

### Statistical analysis

Continuous variables were expressed as median (interquartile range [IQR]) or mean (standard deviation [SD]). Differences between groups for continuous variables were compared using Student’s *t*-test, Wilcoxon signed-rank test, or analysis of variance. Categorical variables were expressed as frequency (*n*) and percentages (%). Chi-square tests or Fisher's exact tests were used to determine differences between categorical variables. Temporal changes in repeated-measures data were investigated by Friedman's test, and effect sizes were determined by the statistic of Kendall's W. Pairwise Wilcoxon signed-rank tests were used to identify differences between two consecutive time points. For the association between factors and binary outcomes, univariable and multivariate logistic models were used to estimate odds ratios (ORs), with 95% confidence intervals (95% CIs). Multivariate logistic models adjusted risk factors including age, sex, smoking, body mass index (BMI), comorbidities (e.g. hypertension, diabetes, cardiovascular disease, Hepatitis B infection, and cancer), and disease severity. R version 4.1.0 was used for all statistical analyses and graphs, and a two-sided *P* value less than 0.05 was considered statistical significance.

## Results

### Baseline characteristics of study participants

The final analysis included 155 survivors. Baseline information for 155 participants was shown in Table 1. The mean age of the patients was 43 (IQR, 34-55) years old, and 74 (47.7%) were female. 19.4% of the participants had comorbidities, including hypertension (13.5%), diabetes (2.6%), cardiovascular disease (1.9%), HBV infection (3.2%), and cancer (1.5%). Among them, 27 (17.4%) patients were classed in the severe group, and 5 (3.2%) patients were admitted to the intensive care unit (ICU) with a median stay time of 15 (5–20) days. Comparison of the clinical characteristics of the patients according to the disease severity, 27 patients in the severe group were older, had higher BMI, and had more comorbidities. During follow-up period, the patient did not receive any treatment for long-term health conditions, such as pharmacological or pulmonary physiotherapy.

**Table 1.** Baseline characteristics.

Variables	All patients	Non-severe patients	Severe patients	*P* value^a^
*N*	155	128	27	
Age, median (IQR), y	43 (34–55)	40 (34–51)	55 (44–62)	0.001
Sex, female, *N* (%)	74 (47.7)	65 (50.8)	9 (33.3)	0.151
Body mass index, mean (SD), kg/m²	23.80 (3.19)	23.40 (3.13)	25.65 (2.84)	0.001
Smoking, *N* (%)	16 (10.9)	13 (10.8)	3 (11.1)	1.000
Comorbidities				
Any, *N* (%)	30 (19.4)	20 (15.6)	10 (37.0)	0.022
Hypertension, *N* (%)	21 (13.5)	15 (11.7)	6 (22.2)	0.254
Diabetes, *N* (%)	4 (2.6)	2 (1.6)	2 (7.4)	0.283
Cardiovascular disease, *N* (%)	3 (1.9)	2 (1.6)	1 (3.7)	1.000
Hepatitis B infection, *N* (%)	5 (3.2)	4 (3.1)	1 (3.7)	1.000
Cancer, *N* (%)	2 (1.3)	1 (0.8)	1 (3.7)	0.776
Hospitalization in ICU, *N* (%)	5 (3.2)	0 (0.0)	5 (18.5)	<0.001
Duration of ICU stay, median (IQR), d	NA	NA	15 (5–20)	NA
Hospitalization period, median (IQR), d	21 (16–28)	20 (16–26)	32 (22–38)	<0.001
Time from symptom to follow-up, median (IQR), d	697 (680–717)	695 (680–716)	715 (684–723)	0.1555

SD, standard deviation; IQR, interquartile range; NA, not available.

^a^ The difference was determined by Student’s *t*-test, Wilcoxon rank sum test, Pearson's Chi-squared test, and Fisher's exact test.

### Long COVID symptom and psychological symptom

**Figure 1.**
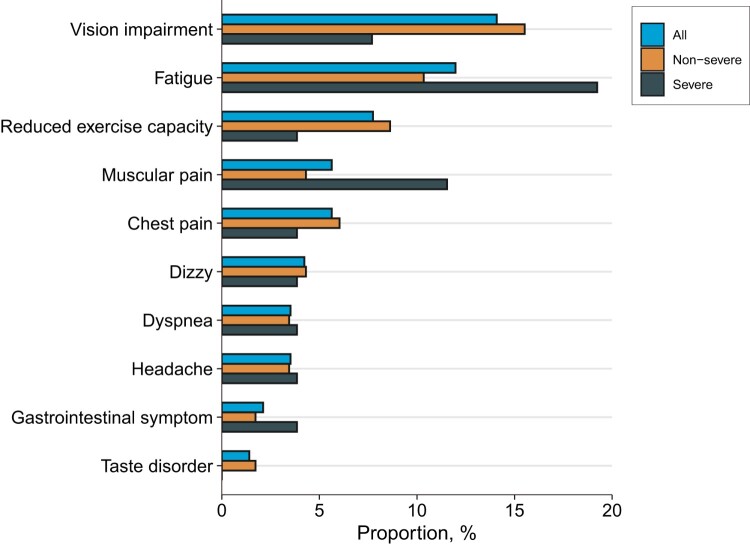
Ten most common symptoms of long COVID at two years.

A total of 143 participants completed the symptom survey to report new and persistent symptoms or any symptoms that were more severe than before the onset of COVID-19. Of these, 52 (36.6%) participants still reported at least one symptom. The most common symptoms were vision impairment (14.1%), fatigue (12.0%), and reduced exercise capacity (7.7%; Figure. 1). Using four psychological questionnaires, 35.0% of participants still presented at least one psychological symptom. The prevalence of anxiety, depression, PTSS, and sleep difficulties were 8.4%, 9.1%, 10.5%, and 27.3%, respectively (Table 2). A comparison of 1-year and 2-year follow-up showed that the number of long COVID symptoms was decreasing, but psychological symptoms were not significantly improved (Table S1).

**Table 2.** Healthy outcomes in patients at 2 years after the infection.

Variables	All patients	Non-severe patients	Severe patients	*P* value^a^
General symptom	142	116	26	
Any, *N* (%)	52 (36.6)	42 (36.2)	10 (38.5)	1.000
Symptom count, mean (SD)	0.69 (1.24)	0.68 (1.23)	0.73 (1.31)	0.854
Psychological symptom	143	117	26	
Any, *N* (%)	50 (35.0)	42 (35.9)	8 (30.8)	0.788
Depression, *N* (%)	13 (9.1)	10 (8.5)	3 (11.5)	0.918
Anxiety, *N* (%)	12 (8.4)	8 (6.8)	4 (15.4)	0.303
PTSS, *N* (%)	15 (10.5)	13 (11.1)	2 (7.7)	0.872
Sleep disorders, *N* (%)	39 (27.3)	32 (27.4)	7 (26.9)	1.000
Respiratory system				
Dyspnea	141	116	25	0.780
mMRC = 0, *N* (%)	123 (87.2)	100 (86.2)	23 (92.0)	
mMRC = 1, *N* (%)	17 (12.1)	15 (12.9)	2 (8.0)	
mMRC = 2, *N* (%)	1 (0.7)	1 (0.9)	0 (0.0)	
Pulmonary function	96	82	14	
FVC < 80% predicted, *N* (%)	4 (4.2)	2 (2.4)	2 (14.3)	0.185
FEV1 < 80% predicted, *N* (%)	10 (10.4)	8 (9.8)	2 (14.3)	0.969
FVC / FEV1 < 80% predicted, *N* (%)	3 (3.1)	3 (3.7)	0 (0.0)	1.000
DLCO < 80%, predicted, *N* (%)	23 (24.0)	20 (24.4)	3 (21.4)	1.000
DLCO / VA < 80%, predicted, *N* (%)	4 (4.3)	2 (2.6)	2 (14.3)	0.205
TLC < 80%, predicted, *N* (%)	3 (3.1)	2 (2.4)	1 (7.1)	0.917
RV < 80% predicted, *N* (%)	3 (3.1)	0 (0.0)	3 (21.4)	0.001
CT scan	146	119	27	
Involvement of the lesions, *N* (%)				0.031
No involvement	72 (49.3)	64 (53.8)	8 (29.6)	
Single lobe	36 (24.7)	29 (24.4)	7 (25.9)	
Bilateral multilobe	38 (26.0)	26 (21.8)	12 (44.4)	
Feature, *N* (%)				
Fibrosis-like lesions	58 (39.7)	43 (36.1)	15 (55.6)	0.100
GGO	20 (13.7)	11 (9.2)	9 (33.3)	0.003
No. of lobes involved, mean (SD)	1.03 (1.30)	0.87 (1.14)	1.74 (1.68)	0.001
Total CT score, mean (SD)	1.08 (1.50)	0.87 (1.18)	2.00 (2.25)	<0.001

PTSS, post-traumatic stress symptoms; FVC, forced vital capacity; FEV1, forced expiratory volume in 1 s; DLCO, diffusing capacity of the lung for carbon monoxide; DLCO/VA, DLCO corrected for alveolar volume; TLC, total lung capacity; RV, residual volume; GGO, ground-glass opacity.

^a^ *P* values were determined with Student’s *t*-test, or Wilcoxon rank sum test, or Chi-squared test.

### Respiratory outcome

Symptoms of dyspnea assessed using the mMRC scale were observed in 18 (12.8%) patients, with 17 (12.1%) having an mMRC of 1 and 1 (0.7%) having an mMRC of 2. Out of 155 participants, 96 patients underwent pulmonary function tests (Table 2). Diffusion dysfunction (23/96, 24.0%) was the most common abnormality. Abnormalities in FVC (4.2%), FEV1 (3.1%), TLC (3.1%), and RV (3.1%) were observed. Comparisons between patients with normal and abnormal DLCO were done. DLCO was not significantly different in severe patients (88.19% of the predicted value) compared to non-severe patients (83.92% of the predicted value, *P* = 0.316). The proportion of diffusing dysfunction in the severe and non-severe group was 24.4% and 21.4%, respectively. Females were more likely to have a DLCO less than 80% of the predicted value at two years after the infection (*P* = 0.044, Table S2).

**Figure 2.**
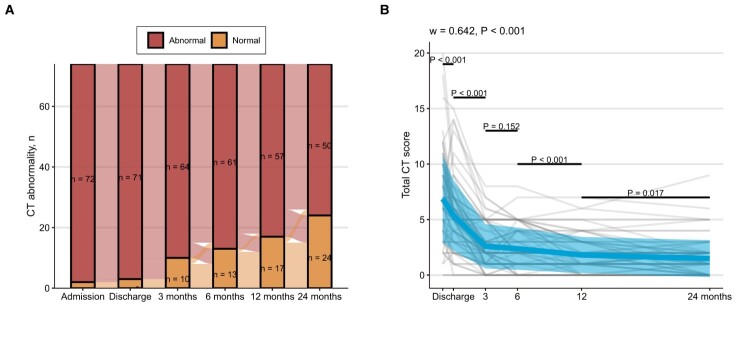
Changes in (A) CT abnormality and (B) CT severity scores over time. The kinetics of CT severity scores at consecutive time points were investigated using Friedman's test (grouping by individual). The Kendall’s W was used as the measure of the Friedman test effect size. The paired Wilcoxon test was used for comparing differences between two consecutive time points. Plots show individual score trajectories as thin grey lines, red lines represent means, and coloured areas represent mean ± standard deviation. The figure shows the number of individuals who completed a series of consecutive CT scans in 74 cases.

Abnormal chest CT scan was observed in 74 of 146 (50.7%) participants. 53.8% of patients in the non-severe group had complete absorb of lung injury, which was significantly higher than 29.6% in the severe group. The predominant pulmonary abnormalities were fibrosis-like lesions (39.7%) and residual GGO (13.7%). Crazy paving, consolidation, and air trapping were not observed. Multivariable analysis suggested that age > 50 years (OR = 1.051, 95% CI: 1.017–1.089; *P* = 0.004) and higher BMI (OR = 1.222, 95% CI: 1.063–1.426; *P* = 0.007) were associated with persistent CT abnormalities (Table S2). In the entire cohort of 74 patients with completed CT data, participants showed progressive improvement in lung involvement over time (Figure 2). In the entire cohort, patients recovered rapidly from discharge to 3 months (3 months vs. discharge, *P* < 0.001), but did not improve significantly at 6 months (6 months vs. 3 months, *P* = 0.152), and gradually recovered from 6 months to 24 months (12 months vs. 6 months, *P* < 0.001; 24 months vs. 12 months, *P* = 0.017). Participants in the severe group had higher baseline CT scores and slower improvement compared to those in the non-severe group (Figure S1).

### Extrapulmonary organ assessment

Two years after the infection, the levels of most laboratory parameters, such as white blood cell count, lymphocyte count, platelet count, lactate dehydrogenase, C-reactive protein, and D-dimer, had returned to normal ranges, and the proportion of patients with these abnormalities had gradually declined from discharge to two years (Table S3). The abnormalities of haemoglobin, creatine kinase, and glucose were commonly seen. Regarding to kidney function, 21.5% of patients had a decrease in eGFR (< 90 mL/min/1.73 m2) and 6.9% had proteinuria (Table 3). The comparison of renal function parameters between the non-severe and severe groups showed a significant difference in the levels of eGFR, Cr, and α1-microglobulin. Older age and male gender were the independent risk factors of decreased eGFR at two years (Table S2). In the dynamic assessment of renal function, eGFR decreased continuously from 3 months to 12 months, and there was no significant difference in eGFR between 12 and 24 months (Figure S2). 15.3% of patients had any kind of liver function test abnormality, with 9.3% of ALT elevation, 2.0% of AST elevation, and 10.7% of GGT elevation, respectively. The GGT levels in the severe group were significantly higher than those in the non-severe group. High BMI was associated with any kind of liver function test abnormalities (Table S2). Liver function tests improved in most patients from hospital discharge to 12 months, and all of ALT, AST, and GGT were significantly elevated between 12 and 24 months (Figure S3, Figure S4).

**Table 3.** The distribution of parameters in kidney and liver function at two years after infection

Variables	All patients	Non-severe patients	Severe patients	*P* value^a^
Kidney function				
Blood detection	149	123	26	
BUN, mean (SD), mmol/L	4.77 (1.30)	4.67 (1.25)	5.21 (1.47)	0.055
Cr, mean (SD), μmoI/L	69.81 (20.23)	67.65 (18.67)	80.00 (24.31)	0.004
eGFR, mean (SD), mL/min/1.73 m2	102.80 (24.98)	105.79 (23.95)	87.24 (24.98)	0.002
eGFR categories, mL/min/1.73 m2, N (%)				<0.001
G1: ≥ 90	117 (78.5)	104 (84.6)	13 (50.0)	
G2: 60–89	28 (18.8)	17 (13.8)	11 (42.3)	
G3a: 45–59	4 (2.7)	2 (1.6)	2 (7.7)	
Urine detection a	130	109	21	
PRO, *N* (%)	9 (6.9)	7 (6.4)	2 (9.5)	0.957
Urinary micro-protein detection	130	109	21	
A1M, mean (SD), mg/L	10.78 (7.80)	10.01 (7.17)	14.78 (9.75)	0.010
B2M, mean (SD), mg/L	0.26 (0.29)	0.25 (0.26)	0.33 (0.41)	0.204
RBP, mean (SD), mg/L	1.80 (6.46)	2.00 (7.04)	0.75 (0.29)	0.420
KAP, mean (SD), mg/L	10.22 (6.38)	9.94 (6.40)	11.64 (6.21)	0.266
LAM, mean (SD), mg/L	4.40 (1.70)	4.44 (1.84)	4.21 (0.60)	0.581
Liver function	150	124	26	
Any LFT abnormalities, *N* (%)	23 (15.3)	16 (12.9)	7 (26.9)	0.132
ALT, mean (SD), U/L	22.37 (15.13)	21.39 (14.19)	27.04 (18.60)	0.084
>40, *N* (%)	14 (9.3)	9 (7.3)	5 (19.2)	0.124
AST, mean (SD), U/L	22.75 (7.64)	22.58 (7.64)	23.54 (7.73)	0.564
>40, *N* (%)	3 (2.0)	2 (1.6)	1 (3.8)	1.000
GGT, mean (SD), U/L	28.86 (41.82)	25.14 (18.12)	46.62 (91.71)	0.017
>49, *N* (%)	16 (10.7)	12 (9.7)	4 (15.4)	0.612
ALB, mean (SD), g/L	46.93 (2.66)	46.93 (2.53)	46.94 (3.25)	0.982
<35, *N* (%)	0 (0.0)	0 (0.0)	0 (0.0)	NA

BUN, blood urea nitrogen; Cr, creatinine; eGFR, estimated glomerular ﬁltration rate; PRO, urine protein; A1M, α1-microglobulin; B2M, β2-microglobulin; RBP, retinol-binding protein; KAP, kappa free light chain; LAM, lambda light chain; LFT, liver function test; ALT, alanine aminotransferase; AST, alkaline phosphatase; GGT, gamma-glutamyltransferase; ALB, albumin; NA, not available.

^a^ *P* values were calculated with Student’s *t*-test or Chi-squared test.

## Discussion

With the ongoing SARS-CoV-2 pandemic, the clinical manifestation and treatment of the acute phase of COVID-19 are now better understood, but data on the long-term effects of COVID-19 are still scarce. This study supports that long COVID is common after SARS-CoV-2 infection and can persist for at least 2 years.

COVID-19 survivors, especially those with severe illness, may experience long-term effects following infection, with symptoms lasting weeks, months, or even years. To date, there is no uniform definition of the long-term health effects of COVID-19. Several terms, such as long COVID or Post-COVID Conditions, Post-COVID-19 Syndrome, and PASC, have been proposed. And the heterogeneity has been observed in long COVID across studies [16]. The estimated prevalence depends on follow-up duration, population and symptoms for defining long COVID. In our follow-up study, more than 30% of participants had persistent symptoms, regardless of disease severity at the acute phase. The previous study in Wuhan reported that 55% of COVID-19 survivors had at least one symptom by two years following infection, and the fatigue or muscle weakness and sleep difficulties were the most frequently reported symptoms [11]. Similarly, the fatigue and sleep difficulties were common in our study; however, the most common symptom was vision impairment, which occurred more frequently in non-severe patients. This may partly be due to a selection bias; one possible reason of vision impairment is that the normal function of eye tissue declines with advancing age. Patients (mean age = 52.7) with vision impairment were significantly older than those (mean age = 46.3) without impairment (*P* = 0.035). However, the long-term effect of COVID-19 on the eye should remain a concern to further studies, as ocular complications have been reported from COVID-19 that may affect the retina and its nerves [17]. The emerging data suggest that people who were vaccinated before SARS-CoV-2 infection were less likely to report long COVID [18]. The prevalence in people with the omicron variant was lower than that in those with the earlier variant [19]. The high prevalence of long COVID symptoms and persistent psychological symptoms highlight the importance of follow-up patients with COVID-19 to explore the long-term effects of this widespread emerging infectious disease and adapt interventions to prevent a possible public health crisis.

The respiratory system is the most affected organ by COVID-19. Our study showed that a considerable subset of participants had impaired lung function for up to two years, with reduced diffusing capacity (24.0%) being the most prominent finding. The prevalence of reduced diffusing capacity we report is in line with the findings of recent reports. For instance, a recent study showed that 35.8% (87 of 243) of patients had impaired DLCO at one year after discharge [5]. Recent data reports that the prevalence of persistent CT abnormalities ranged from 7% to 94% at one year after discharge, and varied by disease severity of enrolling patients in the studies [5,6,20–26]. Pulmonary fibrosis during the recovery stage has raised attention, and chronic fibrosis has been reported in recovery patients who underwent lung biopsy [27]. We found that fibrosis-like lesion was observed in 39.7% of participants at two years. Prevalence of pulmonary fibrosis varies widely across studies and ranges from none to 72% [25,28–30]. Whether fibrosis-like lesion represented true fibrotic lung disease remains uncertain, as it was defined with different radiographic features and lacked histopathologic findings in our and previous studies. Our findings are consistent with a recent study which prospectively evaluated the lung abnormalities by chest CT for 2, 3, 6 months, and one year in 58 patients after the onset of COVID-19, and found progressive radiographic improvement [23]. However, a higher rate of overall improvement of CT scores in the severe group was not observed in our study. Although the trajectory of CT showed that pulmonary involvement in COVID-19 patients significantly improved at two years, the pulmonary lesions were not completely absorbed in over half of the patients yet. Longer follow-up studies are needed to determine the duration of CT and lung function abnormalities.

SARS-CoV-2 infection may affect multiple organs beyond the lungs, including kidneys and livers [31,32]. In this study, abnormal renal function particularly reflected by the decreased eGFR, was still common in patients with COVID-19 at two years after infection. Similar to our findings, another study found that 35% of patients had decreased eGFR (<90 ml/min/1.73 m2) at 6 months after hospitalization for COVID-19 [5]. Our longitudinal study revealed a decline in eGFR between 3 and 12 months of follow-up without an improvement at 24 months. A large-scale study using Veterans Health Administration electronic health records also comprehensively assessed the long COVID in which 30-day survivors of COVID-19 exhibited a higher risk of longitudinal declines in eGFR after the acute phase of the disease [33]. Notably, at present, few articles focus on the long-term impact of COVID-19 on liver function. Consistent with our previous study on the 12-month liver function test, the current finding showed that the elevated GGT was the main marker of liver function abnormality in patients during 2-year follow-up period [34]. In this study, abnormal liver function tests continued to decline after discharge; however, of note, the levels of AST, ALT, and GGT increased significantly at two years. These may reflect the potential long-term effects on multiple organs caused by SARS-CoV-2.

The mechanism that patients develop long COVID symptoms after infection is unclear, which includes viral persistence, SARS-CoV-2 superantigen-mediated activation of the immune system, and autoimmunity [35]. A longitudinal study reveals that risk factors at the time of disease onset, such as pre-existing type 2 diabetes, latent EBV reactivation, SARS-CoV-2 viremia, and specific auto-antibodies, are all associated with specific long-term symptoms [36]. Our study found that specific clinical characteristics, including age, gender, BMI, and disease severity, were associated with health status two years after infection.

The strength of our study is the longitudinal assessment of the health status of patients after mild to critical SARS-CoV-2 infection. Our findings add to the ongoing research on multiple organ effects of COVID-19 by following prospectively over time, with a longer follow-up period. Our study has several limitations. Firstly, all patients were enrolled from one hospital, possibly introducing a selection bias. And some information about the patient's health outcomes is lacking. For instance, we did not assess patients’ cognitive changes. Previous studies have shown that SARS-CoV-2 infection is associated with an increase in the risk of cognitive impairment [37–39]. Secondly, the participants were recruited during the first wave of epidemics in China (January 2020 to April 2020). Long-term manifestations of these patients may differ from those infected with the SARS-CoV-2 variants or received advanced treatments or vaccines. Thirdly, the organ dysfunctions may not be specific to COVID-19 as our study did not include populations without SARS-CoV-2 infection as controls. A recent study found that core symptoms were attributed to COVID-19 in 12.7% of participants [40]. Finally, some patients might have organ abnormalities before the SARS-CoV-2 infection due to a lack of pre-COVID-19 assessments.

Two years after the infection of COVID-19, most patients showed significant improvement in general symptoms and chest CT. However, a considerable proportion of patients had persistent healthy dysfunctions including lung, kidney and liver. Our study provides new insights into the recovery trajectory of patients with COVID-19 after the acute phase.

## Supplementary Material

Supplemental Material
