# Supplementary material for: Healthy outcomes of patients with COVID-19 two years after the infection: a prospective cohort study
Source: Emerg Microbes Infect. 2022 Nov 4;11(1):2680–8. doi: 10.1080/22221751.2022.2133639 (PMC9639507; doi:10.1080/22221751.2022.2133639)
Supplement: Supplemental Material [file TEMI_A_2133639_SM4369.docx]

**Supplementary Material**

**Supplemental Figure S1.** Changes in CT severity scores over time in (A) non-severe group (N = 55) and (B) severe group (N = 19).

The kinetics of CT severity scores at consecutive time points were investigated using Friedman's test (grouping by individual). The Kendall’s W was used as the measure of the Friedman test effect size. The paired Wilcoxon test was used for comparing differences between two consecutive time points. Plots show individual score trajectories as thin gray lines, red lines represent means, and colored areas represent mean +/- standard deviation.

**Supplemental Figure S2.** Changes in (A) eGFR categories and (B) eGFR over time.

The eGFR categories were based on the CKD-EPI formula. The kinetics of eGFR at consecutive time points were investigated using Friedman's test (grouping by individual). The Kendall’s W was used as the measure of the Friedman test effect size. The paired Wilcoxon test was used for comparing differences between two consecutive time points. The figure shows the number of individuals who completed a series of consecutive eGFR in 108 cases. ns, P > 0.05; *, P ≤0.05; **, P ≤0.01; ***, P ≤0.001; ****, P ≤ 0.0001.

**Supplemental Figure S3.** Changes in any kind of liver function tests abnormalities over time.

The plot showed liver function test (LFT) in 112 patients with complete data in the study period. Any kind of LFT abnormality was deﬁned as the levels of ALT, AST, and GGT higher than the upper limit of normal (ULN), or the levels of albumin less than the lower limit of normal (LLN).

**Supplemental Figure S4.** Changes in liver function tests of ALT, AST, GGT, and ALB.

The kinetics of liver biochemistry at consecutive time points were investigated using Friedman's test (grouping by individual). The Kendall’s W was used as the measure of the Friedman test effect size. The paired Wilcoxon test was used for comparing differences between two consecutive time points. The bars represent mean +/- standard deviation. ns, P > 0.05; *, P ≤0.05; **, P ≤0.01; ***, P ≤0.001; ****, P ≤ 0.0001.

**Supplemental Table S1.** Comparison of health outcomes between 1 year and 2 years

| **Variables** | **1 year** | **2 years** | ***P* value ***** |
| --- | --- | --- | --- |
| **General symptom** | 124 | 142 |  |
| Any, N (%) | 64 (51.6) | 52 (36.6) | **0.019** |
| Symptom count, mean (SD) | 1.10 (1.63) | 0.69 (1.24) | **0.022** |
| **Psychological symptom** | 117 | 143 |  |
| Any, N (%) | 39 (34.8) | 50 (35.0) | 1.000 |
| Depression, PHQ-9 > 5, N (%) | 14 (12.5) | 13 (9.1) | 0.501 |
| Anxiety, GAD-7 > 5, N (%) | 10 (8.9) | 12 (8.4) | 1.000 |
| PTSS, PCL-5 > 33, N (%) | 11 (9.8) | 15 (10.5) | 1.000 |
| Sleep disorders, PSQI > 5, N (%) | 28 (25.0) | 39 (27.3) | 0.790 |
| **Respiratory system** |  |  |  |
| **Pulmonary function, N** | 44 | 96 |  |
| FVC < 80% predicted, N (%) | 2 (4.5) | 4 (4.2) | 1.000 |
| FEV1 < 80% predicted, N (%) | 3 (6.8) | 10 (10.4) | 0.713 |
| FVC / FEV1 < 80% predicted, N (%) | 0 (0.0) | 3 (3.1) | 0.578 |
| DLCO < 80%, predicted, N (%) | 15 (34.1) | 23 (24.0) | 0.295 |
| DLCO / VA < 80%, predicted, N (%) | 4 (9.1) | 4 (4.3) | 0.478 |
| TLC < 80%, predicted, N (%) | 5 (11.4) | 3 (3.1) | 0.119 |
| RV < 80% predicted, N (%) | 1 (2.3) | 3 (3.1) | 1.000 |
| **CT scan, N** | 119 | 146 |  |
| Involvement of the lesions, N (%) |  |  | 0.057 |
| No involvement | 44 (35.8) | 72 (49.3) |  |
| Single lobe | 33 (26.8) | 36 (24.7) |  |
| Bilateral multilobe | 46 (37.4) | 38 (26.0) |  |
| No. of lobes involved, mean (SD) | 1.44 (1.47) | 1.03 (1.30) | **0.015** |
| Total CT score, mean (SD) | 1.52 (1.62) | 1.08 (1.50) | **0.022** |

* *P* values were determined with student *t* test, or Wilcoxon rank sum test, or Chi-squared test.

Abbreviations: GAD-7, Generalized Anxiety Disorder 7-item; PHQ-9, Patient Health Questionnaire-9; PTSS, post-traumatic stress symptoms; PCL-5, Posttraumatic Stress Disorder Checklist; PSQI, Pittsburgh Sleep Quality Index; FVC, forced vital capacity; FEV_1_, forced expiratory volume in 1 s; MMEF, maximal mid-expiratory flow; *D*_LCO_, diffusing capacity of the lung for carbon monoxide; *D*_LCO_/*V*_A_, *D*_LCO_ corrected for alveolar volume; TLC, total lung capacity; RV, residual volume.

**Supplemental Table S2.** Risk factors associated with any general symptom, diffusion impairment, radiological abnormalities, decreased eGFR, and any LFT abnormality

|  | **OR (95% CI) *** | ***P* value** |
| --- | --- | --- |
| **Any general symptom** |  |  |
| Age, ≥ 50 y vs. < 50 y | 0.904(0.400-2.000) | 0.804 |
| Sex, male vs. female | 1.034(0.447-2.368) | 0.936 |
| BMI, per unit | 1.107(0.974-1.267) | 0.127 |
| Smoking, yes vs. no | 0.302(0.063-1.091) | 0.090 |
| Any complication, yes vs. no | 1.757(0.687-4.518) | 0.237 |
| Severity, severe vs. non-severe | 0.710(0.254-1.872) | 0.497 |
| **Diffusion impairment** |  |  |
| Age, ≥ 50 y vs. < 50 y | 0.791(0.246-2.418) | 0.685 |
| Sex, male vs. female | 0.274(0.071-0.915) | **0.044** |
| BMI, per unit | 0.920(0.743-1.121) | 0.421 |
| Smoking, yes vs. no | 0.816(0.040-6.093) | 0.861 |
| Any complication, yes vs. no | 1.107(0.253-4.311) | 0.886 |
| Severity, severe vs. non-severe | 1.251(0.231-5.536) | 0.776 |
| **Radiological abnormalities** |  |  |
| Age, ≥ 50 y vs. < 50 y | 1.051(1.017-1.089) | **0.004** |
| Sex, male vs. female | 0.901(0.374-2.134) | 0.814 |
| BMI, per unit | 1.222(1.063-1.426) | **0.007** |
| Smoking, yes vs. no | 0.709(0.190-2.649) | 0.604 |
| Any complication, yes vs. no | 0.714(0.258-1.948) | 0.510 |
| Severity, severe vs. non-severe | 1.253(0.462-3.552) | 0.661 |
| **Decreased eGFR** |  |  |
| Age, ≥ 50 y vs. < 50 y | 1.110(1.054-1.181) | **<0.001** |
| Sex, male vs. female | 9.218(2.563-39.492) | **0.001** |
| BMI, per unit | 0.976(0.798-1.180) | 0.809 |
| Smoking, yes vs. no | 1.073(0.234-4.579) | 0.924 |
| Any complication, yes vs. no | 1.672(0.528-5.135) | 0.372 |
| Severity, severe vs. non-severe | 2.375(0.741-7.582) | 0.141 |
| **Any LFT abnormality** |  |  |
| Age, ≥ 50 y vs. < 50 y | 0.957(0.908-1.005) | 0.089 |
| Sex, male vs. female | 1.930(0.537-7.503) | 0.320 |
| BMI, per unit | 1.392(1.157-1.729) | **0.001** |
| Smoking, yes vs. no | 0.796(0.151-3.341) | 0.766 |
| Any complication, yes vs. no | 0.309(0.042-1.460) | 0.178 |
| Severity, severe vs. non-severe | 2.286(0.614-8.409) | 0.209 |

* The OR were calculated with multivariable logistic model that adjusted all variable.

**Supplemental Table S3.** The distribution of routine laboratory parameter at two years after infection

| **Variables** | **All patients** | **Non-severe patients** | **Severe patients** | ***P* value** * |
| --- | --- | --- | --- | --- |
| N | 150 | 124 | 26 |  |
| White blood cell count, ×10 ^9^/L, mean (SD) | 5.82 (1.33) | 5.83 (1.33) | 5.80 (1.31) | 0.908 |
| < 3.5, N (%) | 0 (0.0) | 0 (0.0) | 0 (0.0) | NA |
| Lymphocyte count, ×10 ^9^/L, mean (SD) | 1.91 (0.54) | 1.90 (0.55) | 1.96 (0.53) | 0.598 |
| < 1.1, N (%) | 5 (3.4) | 5 (4.1) | 0 (0.0) | 0.655 |
| Platelet count, ×10 ^9^/L, mean (SD) | 229.97 (49.52) | 233.59 (50.84) | 212.88 (39.19) | 0.052 |
| < 125, N (%) | 2 (1.3) | 2 (1.6) | 0 (0.0) | 1.000 |
| Hemoglobin, g/L, mean (SD) | 145.09 (14.29) | 144.34 (14.55) | 148.65 (12.65) | 0.163 |
| < 130, N (%) | 20 (13.4) | 19 (15.4) | 1 (3.8) | 0.208 |
| Creatine kinase, U/L, mean (SD) | 124.81 (105.87) | 120.72 (107.82) | 144.60 (95.37) | 0.306 |
| > 190, N (%) | 19 (13.0) | 14 (11.6) | 5 (20.0) | 0.416 |
| Lactate dehydrogenase, U/L, mean (SD) | 172.19 (28.01) | 170.17 (26.39) | 181.96 (33.74) | 0.055 |
| > 250, N (%) | 2 (1.4) | 1 (0.8) | 1 (4.0) | 0.766 |
| C-reactive protein, mg/L, mean (SD) | 1.53 (2.26) | 1.40 (1.82) | 2.18 (3.71) | 0.117 |
| > 6, N (%) | 7 (4.8) | 4 (3.3) | 3 (12.0) | 0.177 |
| Glucose, mmol/L, mean (SD) | 5.41 (0.88) | 5.29 (0.69) | 5.97 (1.33) | **<0.001** |
| > 6.1, N (%) | 14 (9.5) | 10 (8.3) | 4 (15.4) | 0.451 |
| D-dimer, μg/mL, mean (SD) | 0.26 (0.08) | 0.25 (0.07) | 0.28 (0.09) | 0.071 |
| >0.5, N (%) | 2 (1.4) | 1 (0.8) | 1 (4.0) | 0.774 |

* *P* values were calculated with student *t* test or Chi-squared test. The missing data was not shown. NA, not available.
